# The coincidence of IgA nephropathy and Fabry disease

**DOI:** 10.1186/1471-2369-14-6

**Published:** 2013-01-11

**Authors:** Dita Maixnerová, Vladimír Tesař, Romana Ryšavá, Jana Reiterová, Helena Poupětová, Lenka Dvořáková, Lubor Goláň, Michaela Neprašová, Jana Kidorová, Miroslav Merta, Eva Honsová

**Affiliations:** 1 Department of Nephrology, Charles University, Prague, Czech Republic; 2 Institute for Inherited Metabolic Disorders, Charles University, Prague, Czech Republic; 3 Department of Cardiovascular Medicine, Charles University, Prague, Czech Republic; 4 Department of Pathology, First Faculty of Medicine, Charles University, Prague, Czech Republic

**Keywords:** Fabry disease, IgA nephropathy, Alpha-galactosidase A

## Abstract

**Background:**

IgA nephropathy (IgAN) is the most common glomerulonephritis, which may also coexist with other diseases. We present two patients with an unusual coincidence of IgAN and Fabry disease (FD).

**Case presentation:**

A 26 year-old man underwent a renal biopsy in February 2001. Histopathology showed very advanced IgAN and vascular changes as a result of hypertension. Because of his progressive renal insufficiency the patient began hemodialysis in August 2001. By means of the blood spot test screening method the diagnosis of FD was suspected. Low activity of alpha-galactosidase A in the patient’s plasma and leukocytes and DNA analysis confirmed the diagnosis of FD. Enzyme replacement therapy started in July 2004. Then the patient underwent kidney transplantation in November 2005. Currently, his actual serum creatinine level is 250 μmol/l. Other organ damages included hypertrophic cardiomyopathy, neuropathic pain and febrile crisis. After enzyme replacement therapy, myocardial hypertrophy has stabilized and other symptoms have disappeared. No further progression of the disease has been noted.

The other patient, a 30 year-old woman, suffered from long-term hematuria with a good renal function. Recently, proteinuria (2.6 g/day) appeared and a renal biopsy was performed. Histopathology showed IgAN with remarkably enlarged podocytes. A combination of IgAN and a high suspicion of FD was diagnosed. Electron microscopy revealed dense deposits in paramesangial areas typical for IgAN and podocytes with inclusive zebra bodies and myelin figures characteristic of FD. FD was confirmed by the decreased alpha-galactosidase A activity in plasma and leukocytes and by DNA and RNA analysis. Enzyme replacement therapy and family screening were initiated.

**Conclusions:**

Our results emphasize the role of complexity in the process of diagnostic evaluation of kidney biopsy samples. Electron microscopy represents an integral part of histopathology, and genetic analysis plays a more and more important role in the final diagnosis, which is followed by causal treatment.

## Background

IgA nephropathy (IgAN) is the most common glomerulonephritis, which may also coexist with other diseases. Diagnosis of IgAN depends on the demonstration of mesangial IgA-dominant staining (by immunofluorescence or by immunohistochemistry). Weak staining for IgG and/or IgM is also detected in about 50% of cases. Early components of the classical complement pathway, namely C1q is frequently absent. However, C3 is detected in more than 90% of cases of primary IgAN. Microscopic hematuria with or without proteinuria (usually < 2 g/24 h) identify 30–40% of pts with IgAN. The clinical presentation of macroscopic hematuria is commonly provoked by upper respiratory tract infection. Some pts already have renal impairment and hypertension at initial presentation [1].

Fabry disease (FD) is an X-linked disorder of glycosphingolipid catabolism caused by a deficiency of the lysosomal enzyme alpha-galactosidase A. This enzymatic defect leads to a progressive systemic accumulation of glycosphingolipids, mainly globotriaosylceramide, in lysosomes of cells of different tissues. It results in many clinical symptoms such as angiokeratomas, hypohidrosis, neuropathic pains, corneal opacities, renal and cardiovascular diseases. FD manifests primarily in affected hemizygous men, heterozygous females have variable levels of alpha-galactosidase A activity with a wide range of clinical signs [2].

The birth prevalence of FD in the Czech population was calculated to be 0,52 per 100 000 live births (or 1 per 100 000 male live births) and 0,77 per 100 000 live births for hemizygous and heterozygous female, respectively [3]. We present two patients with very rare coincidence of IgAN and FD.

## Case presentation

A 26-year-old man was admitted to our Department of Nephrology for a headache and newly ascertained advanced renal insufficiency (serum creatinine level 606 μmol/L, urea nitrogen 21 mmol/L, creatinine clearance 0.29 mL/s) with blended urinalysis (proteinuria 5.6 g/day, microhematuria – 25/ μl). Any serious diseases were noted at the time of admission to the hospital in February 2001. When he was sixteen he was hospitalized for a headache and uncertain abnormality of urinalysis but after this incident the patient was not examined and followed by the doctors. The patient did not take any regular treatment or addictive drugs, he abstained from alcohol and was a nonsmoker. There was an interesting statement in his family history about his 4-year older brother, who was hemodialysed for four years due to end stage renal disease probably evoked by addictive drugs (without verification by a renal biopsy) and he died at the age of 29 years. His five brothers and two sisters had no symptoms for renal or other diseases. The patient’s father died of prostate cancer at the age of 35 and he had no cardiovascular or renal disease, the patient’s mother suffered from a heart attack and epilepsy.

Physical examination of the patient revealed arterial hypertension (blood pressure was 150/100 mmHg, pulse was 70 beats/min), body temperature was 36.1°C, BMI 25, and no other abnormalities were found by clinical examination.

The levels of renal parameters at the time of admission to the hospital are mentioned above. Ultrasound of abdomen detected bilateral smaller kidney (length 98 mm with an unclear structure). Laboratory results showed serum level of potassium 5.3 mmol/L, hyperphosphataemia 2.1 mmol/L, total protein 67 g/L, albumin 43 g/L, metabolic acidosis (pH 7.2, HCO3 16.8 mmol/L, BE – 6.8 mmol/L), mild anaemia (the level of hemoglobin 125 g/L), mild hyperuricaemia (the level of uric acid 448 mmol/L), dyslipoproteinaemia with an elevation of LDL cholesterol (total cholesterol 7.5 mmol/L, LDL cholesterol 4.8 mmol/L, triglycerides 3.11 mmol/L), results of liver function tests were negative, urinalysis showed a pH 6, microhematuria – 25/ μl, the 24-h protein excretion was 5.6 g/d). Immunology tests were normal (immunoglobulin A, immunoglobulin M, immunoglobulin G, C3 and C4 complement, antinuclear antibodies, antibodies to extractable nuclear antigens, anti-neutrophil cytoplasmic antibodies, immune complexes). The electrocardiogram revealed the signs of left ventricle hypertrophy.

**Figure 1:**
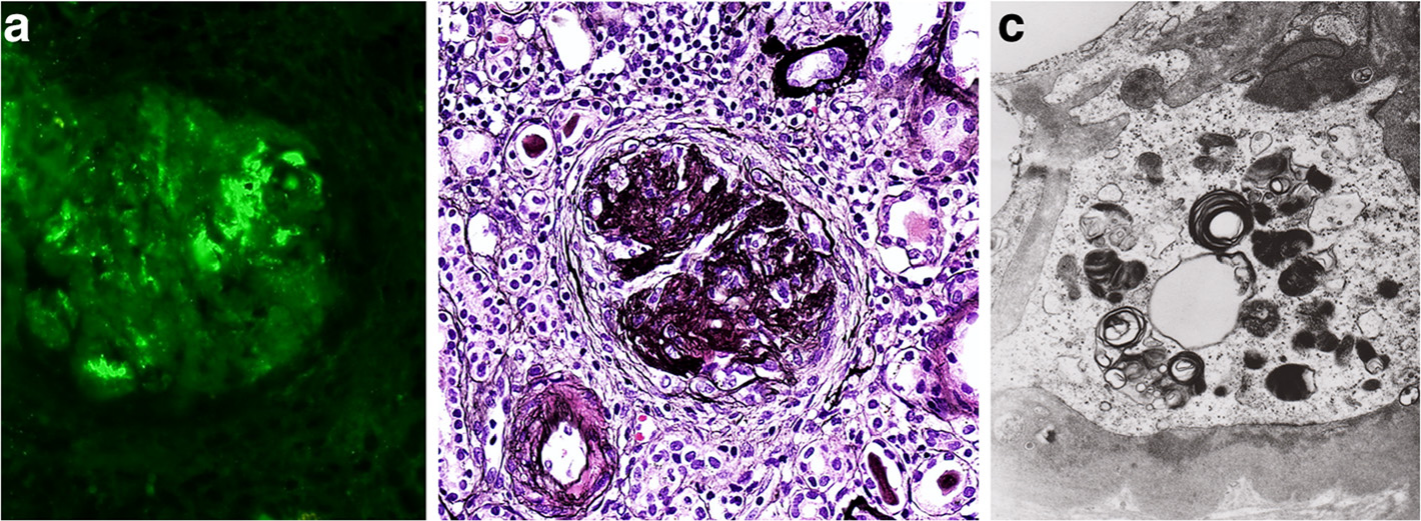
**a. Immunofluorescence with IgA positive staining in the mesangium, which is diagnostic for IgAN. b**. Advanced lesion with diffuse interstitial fibrosis and sclerotic glomerulus. IgAN was confirmed by immunofluorescence. No swollen podocytes indicative of FD are seen (Jones methenamine silver stain; high power field). **c**. Electron microscopy from paraffin embedded tissue. Small dense deposits in mesangial area. Simultaneously lamellated lipid inclusions (so-called myeloid bodies) are shown.

The patient underwent renal biopsy. In a renal biopsy sample, immunofluorescence and light microscopy [Figure 1a,1b] showed an advanced form of mesangioproliferative IgAN with marked scarring and epithelial crescents (in two glomeruli of the 12, the remaining 10 glomeruli were completely sclerotic) and vascular changes as a part of hypertension (electron microscopy was not performed because only sclerotic glomerulus was present in the sample). FD was not identified at that time. Blood pressure was well regulated by means of antihypertensive treatment (angiotensin-converting enzyme inhibitors, beta-blockers, diuretics). The regimen with corticosteroids (Prednisone 0.5 mg/kg) was initiated because of the age of the patient and suspected signs of the disease activity in the renal biopsy sample even though the possible influence of hypertension played a role in the occurrance of epithelial crescents. Subsequently, because of his progressive renal insufficiency the patient started to be hemodialysed in August 2001 (six months after renal biopsy) and corticosteroids were discontinued.

By means of the screening method in dried blood spots in 2003 [4] a diagnosis of FD was suspected. Extensive tests for FD were implemented. Deficient activity of alpha-galactosidase A in the patient’s plasma (patient 0,47 nmol.ml^-1^.h^-1^, normal range 2.4 - 11.3 nmol.ml^-1^.h^-1^) and leukocytes (patient 0.23 nmol.mg^-1^.h^-1^, normal range 30–77 nmol.mg^-1^.h^-1^) confirmed the diagnosis of FD. After the patient’s informed consent was obtained mutation analysis (Sanger sequencing and RFLP method) was performed. We identified a missense mutation c.950 T>C (p.Ile317Thr) in exon 6. This mutation was previously found in an unrelated FD patient [5]. With this knowledge we performed additional electron microscopy from archive paraffin embedded kidney tissue [Figure 1c and additionally showed dense deposits in paramesangial areas as a part of IgAN, and podocytes with inclusions of so-called zebra bodies and myelin figures characteristic of FD. The enzyme replacement therapy started in July 2004 and still continues. The patient underwent kidney transplantation in November 2005. Currently, the actual serum creatinine level is 250 μmol/L without the necessity of dialysis.

**Figure 2:**
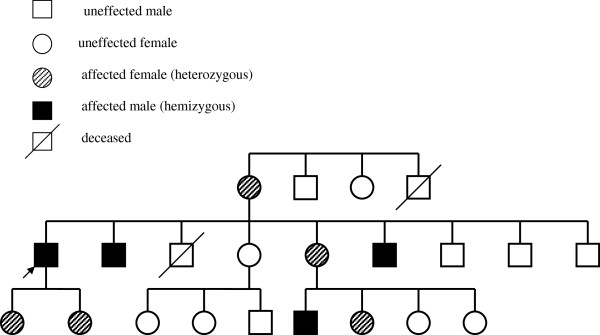
Pedigree of the family of the first patient.

Other organ damages included hypertrophic cardiomyopathy. After the diagnosis of the coincidence of FD and IgAN was assessed we asked the patient about the possible clinical symptoms in his history related to FD and we found two typical signs such as febrile crisis and neuropathic pain in the extremities. Typical symptoms of FD – angiokeratomas and corneal changes – were not observed in this patient. Nevertheless, other findings (febrile and painful crisis, neuropathy, hypertrophic cardiomyopathy and Fabry nephropathy) supported the diagnosis of FD. On enzyme replacement therapy myocardial hypertrophy has stabilised, febrile crisis and neuropathic pain in the extremities have disappeared. To date, no other progression of the disease has been noted. After the informed consent was obtained, mutation analysis of the GLA gene was performed in 12 relatives, using the direct sequencing. Other 8 relatives were newly diagnosed with FD [3 hemizygous and 5 heterozygous, Figure 2].

**Figure 3:**
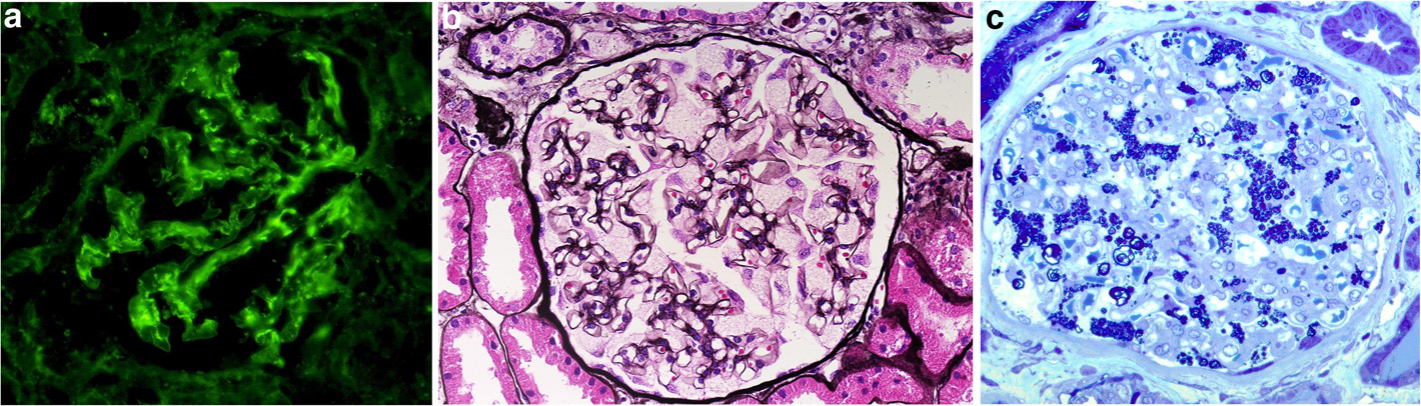
**a. Positive mesangial IgA staining in immunofluorescence (high power field). b**. Light microscopy - glomerulus with remarkably enlarged podocytes with many vacuoles in their cytoplasm. Swollen lacy cytoplasm of podocytes is indicative of FD (Jones methenamine silver stain; high power field). Please note, diagnostic IgA deposits in mesangium were shown in immunofluorescence. **c**. Semithin section stained with toluidin blue showed characteristic “blue inclusions” mainly in podocytes. These features together with large podocytes with vacuolated cytoplasm in light microscopy represent a key to the morphological diagnosis of FD.

**Figure 4:**
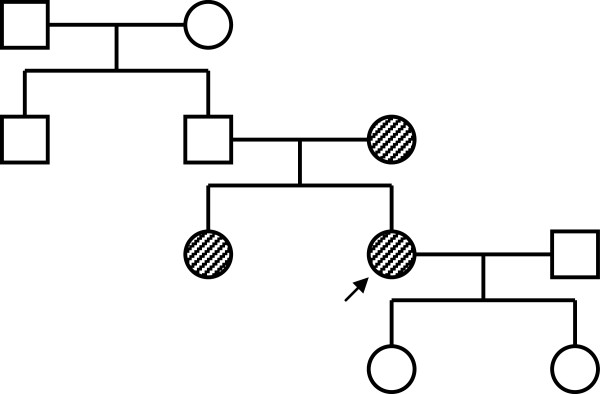
Pedigree of the family of the second patient (the relatives of the proband’s mother refused the genetic analysis).

The second, a 30-year-old woman, suffered from long-term hematuria with a good renal function. Due to increasing proteinuria (2.6 g/day) appeared and a renal biopsy was performed. Histopathology showed IgAN with remarkably enlarged podocytes. IgAN and a high suspicion of a combination with FD was diagnosed by evaluation using immunofluorescence and light microscopy [Figure 3a, 3b. Electron microscopy [Figure 3c, semithin section] revealed dense deposits in paramesangial areas typical of IgAN and podocytes with inclusive zebra bodies and myelin-like bodies characteristic of FD. The activity of alpha-galactosidase A in plasma was within the range of control values (3.3 nmol.ml^-1^.h^-1^, normal range 2.4 - 11.3 nmol.ml^-1^.h^-1^) and was decreased in leukocytes (26 nmol.mg^-1^.h^-1^, normal range 30–77 nmol.mg^-1^.h^-1^). The diagnosis of FD was confirmed by the identification of the *GLA* gene mutation c.1085C>T (p.Pro362Leu). This mutation was previously described in an unrelated FD patient [5]. Mutation analysis was performed after the informed consent was obtained. The mutation analysis also revealed the diagnosis of FD in the patient’s mother and sister (both heterozygous) with the same mutation [Figure 4. Subsequent examination of our patient showed corneal changes (cornea verticillata). Other organ defects were not proved. Enzyme treatment was initiated recently, but we still can not assess the effect of enzyme replacement treatment (the short duration of enzyme treatment – only one month).

## Discussion

IgAN belongs to the major primary glomerulonephritides worldwide [6,7] but it may also occur as a secondary nephropathy as noted above.

FD is an X-linked disease that manifests predominantly in affected hemizygous men. Heterozygous females may have various clinical signs from asymptomatic to severe depending on X-inactivation status [8]. The coexistence of FD with other immune diseases such as rheumatoid arthritis [9], systemic lupus erythematosus [9-13] and celiac sprue [14,15] has been noticed in the literature. FD has also been reported with different nephropathies such as granulomatous interstitial nephritis [16], focal segmental sclerosis [17] and necrotising, crescentic glomerulonephritis [18].

Six reports of the coincidence of IgAN and FD have been published. Three of the reports included three heterozygous women with proteinuria and hematuria, normal renal function and without others significant clinical manifestation of FD [19-21]. The fourth report described a 28 year-old man from Japan with very low alpha-galactosidase A activity and low activity of this enzyme in the patient’s mother [22]. The fifth report referred to two adolescent sisters heterozygous for FD inherited from their father with the rapidly progressing proteinuria in the nephrotic range, glomerular hematuria and the typical signs of FD [2]. The last case report concerned a 22 year-old Japanese man with proteinuria (1.5 g/day) and normal serum creatinine level, with hypohidrosis and neuralgia with fever [23].

Four case reports of the coexistence of IgAN and FD come from Japan. It is claimed that there are some differences in prevalence of IgAN among different ethnicities [24,25]. IgAN seems to be observed more frequently in some Asian countries (Japan and China) than in Europe. This difference could be partly explained by different renal biopsy policy because the patients with isolated microscopic hematuria are indicated to renal biopsy more frequently in Japan in comparison with Europe or America.

The pathogenesis of IgAN has not yet been still completely elucidated. It is hypothesized that aberrantly glycosylated galactose-deficient forms with terminal GalNAc or sialylated GalNAc in the hinge region of IgA1 molecules are supposed to react with naturally occuring anti-IgG and anti-IgA1 antibodies and immune complexes are formed [24]. An elevated serum galactose-deficient IgA1 level was demonstrated to be antecedent to disease but IgA1 glycosylation abnormalities are not sufficient to cause IgAN, additional environmental and genetic cofactors are probably required for an activation of immune complexes and renal injury [24,26,27]. Recent studies showed that the IgA-CIC, composed of galactose-deficient IgA1 complexed with antiglycan antibodies, are bound to mesangial cells more efficiently than uncomplexed IgA [26,27]. Thus, they play a role in the pathogenesis of IgAN. Also familial forms of IgAN [28] have been described. A linkage of IgAN to chromosomes 6q22-q23, 2, 3, 4 and 17 has been demonstrated [29].

The detection of a high level of autoantibodies has been found in patients with FD [10]. However, specific link between IgAN and FD has not yet been disclosed. It has been suggested that glycosphingolipids accumulating in FD continuously and chronically stimulate the immune system and induce an autoimmune reaction [30]. Remarkably, the glycolipid structure of globotriaosylceramide [31] resembles nephritogenoside [32], which causes progressive experimental nephritis similar to IgAN. Thus, some patients with FD are prone to the development of IgAN which may be determined by different genetic and/or environmental factors. We can not also excluded that two separate Mendelian traits (FD and IgAN) were segregating in the described families as was already reported [2]. The specific relationship between IgAN and FD needs to be revealed by further studies.

Interestingly, the 57-year-old mother of our second reported patient had no clinical symptoms compared to her 32 year-old daughter although they had genetically proved the same mutation. It has been reported that family members with FD with the same mutation may have considerably different phenotypes owing to the influence of additional modifier genes and/or environmental factors [33].

As we mentioned, the suspicion of Fabry nephropathy can usually be determined by light microscopy due to enlarged hypertrophic podocytes (only one report confirmed ultrastructural findings from electron microscopy as the first signs suggestive of FD - 21). Nevertheless, immunofluorescence and light microscopy of the renal biopsy sample in our first reported patient did not show features of Fabry nephropathy. Enlarged podocytes with vacuolated cytoplasms in light microscopy and also blue bodies in semithin sections (Figure 3c) represent a key to the diagnosis of FD. However, in advanced stages of various renal diseases, the majority of glomeruli are sclerotic and in the remaining glomeruli with large segmental sclerotic lesions are almost no podocytes, and therefore this diagnostic marker disappears. We were unable to recognize the features of FD in light microscopy and/or in semithin sections even retrospectively in this case. On the basis of a positive “blood spot screening” test, we performed additional electron microscopy from archive paraffin embedded tissue and the result showed the coincidence of IgAN and FD. This case emphasized the important role of electron microscopy in renal pathology even in such a common disease as IgAN. We would like to emphasize that average age of end-stage renal disease in FD patients is around 40 to 50 years. Two reported patients are young, the hemizygous male is 26 years old and the heterozygous female is 30 years old. The coincidence of two diseases of IgAN and FD evidently may accelerate the progression of renal function decline and the combination of IgAN and FD in young patients should raise our awareness of possible differential diagnosis.

The signs of active leasions connected with IgAN (epithelial crescents in two glomeruli of a total of 12) were approved at the time of renal biopsy in our first patient therefore in addition to the inhibitors of angiotensin converting enzyme the immunosuppressive treatment with corticosteroids were initiated (regarding the possibility of the influence of epithelial crescents by hypertension). With respect to grave vascular hypertensive changes, glomerular sclerosis in association with extensive tubulointerstitial scarring (65%), deposits IgA in the mesangium, and typical figures characteristic of FD as were mentioned, it is not possibly to clearly say what was the main cause of the end stage renal disease in this young 26 year-old man. We suppose it was the combination of IgAN, FD and severe vascular changes. We want to emphasize the possibility of initiating of enzyme replacement therapy, which has stabilized myocardial hypertrophy and other symptoms have disappeared. Several reports have unequivocaly documented clinical benefits of enzyme replacement therapy in FD [34,35].

## Conclusion

We have reported a rare coincidence of two diseases IgAN and FD in two Czech patients. Only two case reports of the coincidence of IgAN and FD have been published in Caucasians [2,20]. The precise pathogenesis of the coincidence of these two entities needs to be elucidated by further studies. Nevertheless, our results emphasize the role of complexity in the process of diagnostic evaluation of kidney biopsy samples. Although enlarged podocytes in light microscopy can lead to suspicion of a diagnosis of FD, in advanced cases where glomeruli are predominantly sclerotic this feature can be invisible. In such cases electron microscopy represents the key to diagnosis. Genetic analysis plays an important role in the final diagnosis and enables causal enzyme replacement treatment. Diagnosis of FD in early stages is often very difficult. Therefore, screening of newborns of FD could be considered a subject for future discussion.

## Consent

Written informed consents were obtained from the patients for publication of this Case report. A copy of the written consents is available for review by the Editor of this journal.

## Competing interests

The authors declare that they have no competing interests.

## Authors’ contributions

DM - has made substantial contributions to conception and design of the article, interpretation of data. DM has been involved in revising and drafting the manuscript. VT - has made substantial contributions to design of the article and has given final approval of the version to be published. HP, LD - carried out the genetic analysis and enzyme analysis of alpha-galactosidase A. RR, MM –performed the renal biopsy of the patients and contributed to design of the article and interpretation of data. JR, JK, MN – have contributed to conception, acquisition of clinical data and interpretation of data. LG – performed cardiology examination of the patients and helped with design of the article. EH - has prepared histological evaluation of the cases, has made histological images and substantial contributions to design of the article. EH has been greatly involved in revising the manuscript and has given final approval of the version to be published. All authors read and approved the final manuscript.

## Pre-publication history

The pre-publication history for this paper can be accessed here:

http://www.biomedcentral.com/1471-2369/14/6/prepub
